# Study of myopia progression and risk factors in Hubei children aged 7–10 years using machine learning: a longitudinal cohort

**DOI:** 10.1186/s12886-024-03331-x

**Published:** 2024-03-01

**Authors:** Wenping Li, Yuyang Tu, Lianhong Zhou, Runting Ma, Yuanjin Li, Diewenjie Hu, Cancan Zhang, Yi Lu

**Affiliations:** 1 Department of Ophthalmology, Renmin Hospital of Wuhan University, 238 Jiefang Road, 430060 Wuhan, China; 2 Department of Informatics, University of Hamburg, Hamburg, Germany

**Keywords:** Myopia progression, Refractive status, Children, Risk factors, High myopia, Machine learning

## Abstract

**Background:**

To investigate the trend of refractive error among elementary school students in grades 1 to 3 in Hubei Province, analyze the relevant factors affecting myopia progression, and develop a model to predict myopia progression and the risk of developing high myopia in children.

**Methods:**

Longitudinal study. Using a cluster-stratified sampling method, elementary school students in grades 1 to 3 (15,512 in total) from 17 cities in Hubei Province were included as study subjects. Visual acuity, cycloplegic autorefraction, and height and weight measurements were performed for three consecutive years from 2019 to 2021. Basic information about the students, parental myopia and education level, and the students’ behavioral habits of using the eyes were collected through questionnaires.

**Results:**

The baseline refractive errors of children in grades 1 ~ 3 in Hubei Province in 2019 were 0.20 (0.11, 0.27)D, −0.14 (−0.21, 0.06)D, and − 0.29 (−0.37, −0.22)D, respectively, and the annual myopia progression was − 0.65 (−0.74, −0.63)D, −0.61 (−0.73, −0.59)D and − 0.59 (−0.64, −0.51)D, with the prevalence of myopia increasing from 17.56%, 20.9%, and 34.08% in 2019 to 24.16%, 32.24%, and 40.37% in 2021 (Χ^2^ = 63.29, *P* < 0.001). With growth, children’s refractive error moved toward myopia, and the quantity of myopic progression gradually diminished. (F = 291.04, *P* = 0.027). The myopia progression in boys was less than that in girls in the same grade (*P* < 0.001). The change in spherical equivalent refraction in myopic children was smaller than that in hyperopic and emmetropic children (F = 59.28, *P* < 0.001), in which the refractive change in mild myopia, moderate myopia, and high myopia children gradually increased (F = 73.12, *P* < 0.001). Large baseline refractive error, large body mass index, and high frequency of eating sweets were risk factors for myopia progression, while parental intervention and strong eye-care awareness were protective factors for delaying myopia progression. The nomogram graph predicted the probability of developing high myopia in children and found that baseline refraction had the greatest predictive value.

**Conclusion:**

Myopia progression varies by age, sex, and myopia severity. Baseline refraction is the most important factor in predicting high myopia in childhood. we should focus on children with large baseline refraction or young age of onset of myopia in clinical myopia prevention and control.

**Supplementary Information:**

The online version contains supplementary material available at 10.1186/s12886-024-03331-x.

## Introduction

In recent years, the prevalence of myopia has been increasing worldwide and has gradually become a global public problem of great concern [1]. There are significant ethnic and geographic differences in the distribution of myopia prevalence [2]. East and Southeast Asians have the highest prevalence, such as China (37.7%) [3], South Asians have a much lower rate, such as India (7.5%) [4], black Africans have the lowest prevalence (4.7%) [5], and white Europeans have intermediate prevalence, such as Norway (13.4%) [6], Germany (11.4%) [7], and Ireland (19.9%) [8]. The prevalence of myopia in East Asians has increased by 23% over the past decade, with a slow increase in the prevalence of myopia in South Asians and minimal change in the prevalence of myopia in whites [9]. It is expected that by 2050, the global myopic population will reach 4.76 billion, and the population with high myopia will reach 938 million, accounting for nearly 50% and 10% of the world’s population, respectively [10].

Myopia is a multifactorial disease with a combination of genetic and environmental factors. Many studies have presented evidence regarding the risk factors for the onset and progression of myopia, such as near work, outdoor activities, excessive use of electronic devices, and parental myopia [11]. However, among the above factors, only the causal relationship between education and outdoor activity time and the occurrence of myopia in school-aged children has been confirmed [12], and other factors still need to be further validated by high-quality cohort studies and clinical randomized trials. Since there are also associations between the various factors affecting myopia, for example, an increase in time spent on electronic screens is often accompanied by an increase in near-work and a decrease in time spent outdoors, there are limitations when traditional statistical methods often fail to identify covariate covariance and possible confounders [13]. Therefore, we need to explore new methods to reduce the influence of confounders, identify covariates, and measure the magnitude and importance of interactions between variables.

In this study, we conducted eye examinations and questionnaires on related factors for primary school students in grades 1–3 in Hubei Province, China, for 3 consecutive years (2019–2021), aiming to explore risk factors affecting myopia progression and to construct a personalized model to predict the probability of a child developing high myopia. This study will provide a reference for the development of myopia prevention and control strategies for adolescents.

## Methods

### Study population and sampling

Randomized stratified whole cluster sampling was used in this study. Hubei Province has 17 cities, including 12 prefecture-level cities, 3 directly administered cities, 1 autonomous prefecture, and 1 forested area. The whole group of grade 1–3 students from 2 elementary schools in each city was randomly included in the study. We came to schools for vision and refractive examinations every year, and they are followed closely for three years. Children with ocular diseases affecting vision, such as glaucoma, keratoconus, fundus lesions, strabismus, or a history of ocular surgery, as well as those who were using atropine eye drops or keratoplasty lenses, were excluded.

In the first year (2019), a total of 17,137 students in grades 1 to 3 in Hubei Province were sampled. A total of 15,512 students completed vision and refractive examinations in that year, and 14,213 (82.94%) valid questionnaires matching the students’ information were recovered. The number of students in the second and third years of follow-up was 13,568 and 12,766, respectively, and the response rates in 2019, 2020, and 2021 were 90.52% (15,512/17,137), 87.47% (13,568/15,512), and 94.09% (12,766/13,568), respectively. There was no significant difference in demographic characteristics between participants who completed the 3-year follow-up and those who were dislodged (*P* = 0.841).

### Ethics, consent, and permissions

The study was approved and consented to by the Clinical Ethics Research Committee of Renmin Hospital of Wuhan University(WDRY2020-K211), following the tenets of the Declaration of Helsinki. The purpose of the study and the examination procedure was explained in detail to the students and their parents or legal guardians before the study began. A written agreement for informed consent was obtained from at least one parent, and verbal consent was obtained from each of the examined children at the time of the examination.

### Ocular examination

Visual acuity(VA) was measured at a distance of 5 m using the Standard Logarithmic Visual Acuity Chart (National Standard of the People’s Republic of China, GB11533-2011). VA was converted to logMAR for analysis.

Cycloplegia was achieved by instilling at least five drops of 1% cyclopentolate in intervals of 5 min before obtaining autorefraction measurements (TOPCON RM-8800). During the refractometry, each eye was measured at least three times, and the mean was taken for statistical analysis. The spherical equivalent refraction (SE) was calculated as the spherical value of the refractive error plus half of the cylindrical value. Hyperopia was SE >−0.50 D and < + 0.50 D in both eyes; hyperopia was SE ≥ + 0.50 D in any eye; myopia was SE ≤−0.50 D in any eye; mild myopia was SE >−3.00 D and ≤−0.50 D in any eye; moderate myopia was SE >−6.00 D and ≤−3.00 D in any eye; and high myopia was SE ≤−6.00 D in any eye. The annual refractive change was defined as the total refractive change divided by the months of follow-up and multiplied by 12, with negative values indicating myopia progression. For example, if the total refractive change is −1.50 D over a 36-month follow-up period, then the annual refractive change is −0.50 D (−1.50 D/36 × 12).

### Body index measurement

All subjects removed their shoes and hats when measuring height and weight. Height was recorded in centimeters (cm), and weight was recorded in kilograms (kg). Body mass index (BMI) was calculated as weight/height and recorded in kilograms per square meter (kg/m2). Based on the 2000 Centers for Disease Control and Prevention (CDC) growth icons of BMI percentile for each gender and age group [14], students were categorized into four groups: slim (BMI < 5th percentile), normal weight (5th percentile ≤ BMI < 85th percentile), overweight (85th percentile ≤ BMI < 95th percentile), and obese (BMI ≥ 95th percentile).

All examinations were performed by trained ophthalmologists or optometrists following a standard study protocol. To ensure data quality, 5% of the students were randomly selected for repeat measurements of visual acuity, refraction, height, and weight. If the error between the two tests exceeded the permissible thresholds (visual acuity 0.1 log MAR, refraction 0.5 D, height 0.1 cm, weight 0.1 kg), corrective measures (e.g., additional training) were taken to improve the quality of the data.

### Questionnaire survey

The questionnaires included general information about the students, which included age, gender, whether they were born prematurely, their eye habits, parents’ myopia condition, parents’ education level, daily outdoor time, daily near work time, time spent on electronic devices each day, frequency of eating sweets, and parents’ knowledge of myopia (The questionnaire is included as “Appendix 1”. ). The questionnaires were uniformly distributed to the students and their parents before the examination and were completed by students and parents.

### Statistical analysis

The data were analyzed using the SE for the worst eye of each student. A database was created using Epi Data 3.1, and after data were entered in a double-blind manner and checked for errors, the data were analyzed using the Statistical Package for IBM Social Science Programs V25.0 (SPSS, Chicago, IL, USA) and R software (version 4.0.3, https://www.r-project.org/). Descriptive statistics were expressed as the mean ± standard deviation (mean ± SD) for continuous variables and as the rate (%) for categorical variables. Multifactor linear regression was used to analyze the relationship between individual baseline characteristics and environmental factors (Table 1) and changes in students’ SE. Differences were considered significant at *P* < 0.05 with a confidence interval (CI) of 95%.

**Table 1:** Variable assignments for environmental factors related to myopia

Variable	0	1	2	3
Duration of reading and writing each day	<1 h	1 ~ 2 h	2 ~ 3 h	≥ 3 h
Parents’ education level	Junior middle school equivalent or less	Senior middle school equivalent (Technical school)	Undergraduate degree (Junior college/college)	Postgraduate degree (Masters, PhD)
Duration of electronic devices	<1 h	1 ~ 2 h	2 ~ 3 h	≥ 3 h
Duration of outdoor activity each day	<1 h	1 ~ 2 h	2 ~ 3 h	≥ 3 h
Most frequently used electrical device	television	computer	phone	tablet
Whether to rest after a period of continuous reading	no	yes		
Whether to check vision every six months	no	yes		
Parents’ knowledge of vision care	Exactly not	A little	Some	Very well
Supervision of children’s vision protection	never	sometimes	often	always
Whether to wear glasses for children after myopia	no	yes		
Parents’ awareness of the hazards of myopia	light	moderate	heavy	severe
Whether the child piddle	no	yes		
Frequency of eating sweets or carbonated beverages	never	once or twice a week	3 ~ 5 times a week	Every day

Multiple machine-learning approaches were employed in this work to estimate the annual myopia progression in children from year to year, such as the decision tree algorithm. 90% of the data, randomly selected, were defined as the internal validation group and the rest as the external validation group. In the internal validation group, fivefold cross-validation (80% for training and 20% for validation) was used to tune the parameters and construct an optimal machine-learning model. Then, the model was applied to the external validation group, the receiver operating characteristic (ROC) curves of several machine-learning approaches were calculated, and the model with the best prediction effect was selected to plot its calibration curve (CC) and decision curve analysis (DCA).

## Results

### Baseline characteristics of the study population

The baseline demographic information of the study population is presented in Table 2. The mean age of the first, second, and third grades in the 2019 baseline survey was 7.31 ± 0.46, 8.41 ± 0.49, and 9.26 ± 0.45 years (F = 19382.45, *P* < 0.001). There was no statistically significant difference in the sex ratio among the three grades (X2 = 6.796, *P* = 0.033). The mean equivalent spherical lenses were 0.20 (0.11, 0.27) D in grade 1, −0.14 (−0.21, 0.06) D in grade 2, and − 0.29 (−0.37, −0.22) D in grade 3 (F = 1253.37, *P* < 0.001). The myopia rates in the three grades were 17.56%, 20.90%, and 34.08%, respectively (X2 = 1169.77, *P* < 0.001).

**Table 2:** Baseline characteristics of the subjects (*n* = 12,766)

Variable	All	Grade 1	Grade 2	Grade 3	F/X2	P
number	12,766	4509	4034	4223		
age	8.30 ± 0.94	7.31 ± 0.46	8.41 ± 0.49	9.26 ± 0.45	19382.45	<0.001
Male (%)	7252 (56.80)	2621 (58.1)	2399 (56.8)	2232 (55.3)	6.796	0.033
height (cm)	130.95 ± 9.54	124.58 ± 8.33	131.70 ± 7.77	137.05 ± 7.89	2666.47	<0.001
weight (kg)	27.01 ± 7.36	24.50 ± 5.65	26.67 ± 6.71	30.03 ± 8.42	688.84	<0.001
BMI (kg·m-2)	15.71 ± 3.55	15.37 ± 3.49	15.79 ± 3.21	15.93 ± 3.92	28.17	<0.001
SE (D)	-0.17 (-0.28, 0.11)	0.20 (0.11, 0.27)	-0.14 (-0.21, 0.06)	-0.29 (-0.37, -0.22)	1253.37	< 0.001
Number of myopia (%)	3074 (24.08)	792 (17.56)	843 (20.90)	1439 (34.08)	1169.77	<0.001

### Characteristics of change in refractive error

Table 3 illustrates the overall refractive status of grades 1–3 in Hubei Province for three years. The mean SE for all students was − 0.13 (−0.58, 0.27) D in 2019, −0.72 (−1.18, −0.54) D in 2020, and − 1.27 (−1.51, −0.96) D in 2021, with the overall refractive status progressing toward myopia over time (F = 13.97, *p* < 0.001). The prevalence of myopia also increased from 24.08% in 2019 and 28.13% in 2020 to 30.54% in 2021 (X2 = 63.29, *P* < 0.001).

**Table 3:** Refractive status and prevalence of myopia in grade 1 to 3 students in Hubei Province from 2019 to 2021

		Spherical Equivalent Refraction				Prevalence of Myopia			
		1st visit	2nd visit	3rd visit	*P*	1st visit	2nd visit	3rd visit	*P*
All		-0.13 (-0.58, 0.27)	-0.72 (-1.18, -0.54)	-1.27 (-1.51, -0.96)	< 0.001	24.08%	28.13%	30.54%	< 0.001
Grade 1 (7 ~ 8 y)	Total	0.20 (0.17, 0.27)	-0.48 (-0.53, -0.44)	-0.73 (-1.02, -0.94)	< 0.001	17.56%	19.31%	24.16%	< 0.001
	Male	0.32 (0.23, 0.41)	-0.41 (-0.52, -0.37)	-0.68 (-0.74, -0.61)	< 0.001	15.37%	17.47%	21.49%	< 0.001
	Female	0.16 (0.12, 0.26)	-0.56 (-0.64, -0.49)	-0.84 (-0.89, -0.75)	< 0.001	19.26%	21.35%	26.74%	< 0.001
Grade 2 (8 ~ 9 y)	Total	-0.44 (-0.04, 0.02)	-0.69 (-0.69, -0.62)	-1.06 (-1.10, -1.01)	< 0.001	20.90%	29.65%	32.24%	< 0.001
	Male	-0.36 (-0.42, -0.31)	-0.58 (-0.64, -0.52)	-0.95 (-1.04, -0.87)	< 0.001	18.27%	27.74%	29.76%	< 0.001
	Female	-0.52 (-0.63, -0.47)	-0.75 (-0.80, -0.68)	-1.14 (-1.19, -1.05)	< 0.001	23.83%	30.94%	35.85%	< 0.001
Grade 3 (9 ~ 10 y)	Total	-0.65 (-0.33, -0.26)	-1.00 (-1.04, -0.96)	-1.41 (-1.52, -1.37)	< 0.001	34.08%	37.97%	40.37%	< 0.001
	Male	-0.54 (-0.61, -0.49)	-0.84 (-0.92, -0.78)	-1.32 (-1.41, -1.27)	< 0.001	30.53%	34.29%	38.54%	< 0.001
	Female	-0.74 (-0.82, -0.65)	-1.15 (-1.23, -1.06)	-1.50 (-1.56, -1.43)	< 0.001	36.92%	39.86%	43.91%	< 0.001

The mean SE of first, second, and third graders in 2019 were 0.20 (0.17, 0.27) D, −0.44 (−0.04, 0.02) D, and − 0.65 (−0.33, −0.26) D, respectively. The refractive status shifted from hyperopia to myopia as the grade level increased (F = 451.29, *P* < 0.001). For the same grade, the SE gradually decreased and the myopia rate gradually increased during the three surveys (*P* < 0.001). The SE of boys was greater than that of girls in the same grade during the follow-up, their refractive status was more farsighted (all *P* < 0.001), and their prevalence of myopia was lower (all *P* < 0.001).

The mean change in SE in 2020 was − 0.65 (−0.74, −0.63) D, −0.61 (−0.73, −0.59) D, and − 0.59 (−0.64, −0.51) D for first, second, and third grade respectively. The yearly refractive change significantly reduced as the grade increased. (F = 21.04, *P* = 0.027). The change in SE was consistently smaller for boys than for girls in the same grade, and the progression of myopia was relatively slow (*P* < 0.001) (Table 4).

**Table 4:** Annual refractive change in children from grade 1 to 3 in Hubei Province

		Annual SE change in 2020	Annual SE change in 2021	t	*P*
All		-0.68 (-0.79, -0.57)	-0.65 (-0.78, -0.53)	7.96	< 0.001
Grade 1 (7 ~ 8 y)	Total	-0.65 (-0.74, -0.63)	-0.61 (-0.69, -0.46)	17.85	< 0.001
	Male	-0.59 (-0.65, -0.51)	-0.51 (-0.64, -0.43)	8.41	< 0.001
	Female	-0.70 (-0.79, -0.63)	-0.64 (-0.71, -0.55)	7.39	0.005
Grade 2 (8 ~ 9 y)	Total	-0.61 (-0.73, -0.59)	-0.58 (-0.62, -0.38)	14.58	< 0.001
	Male	-0.57 (-0.64, -0.51)	-0.52 (-0.63, -0.47)	11.32	< 0.001
	Female	-0.68 (-0.74, -0.52)	-0.63 (-0.69, -0.58)	13.39	< 0.001
Grade 3 (9 ~ 10 y)	Total	-0.59 (-0.64, -0.51)	-0.60 (-0.67, -0.44)	21.27	< 0.001
	Male	-0.52 (-0.65, -0.47)	-0.51 (-0.62, -0.48)	12.74	< 0.001
	Female	-0.65 (-0.74, -0.57)	-0.67 (-0.75, -0.61)	15.95	< 0.001

There were significant differences in the annual refractive change among students with different refractive statuses (F = 11.50, *P* < 0.001) (Table 5). In the 2020 survey, the annual refractive error of myopic children [−0.68 (−0.74, −0.57) D] was smaller than that of emmetropic children [−0.49 (−0.52, −0.46) D] (F = 59.28, *P* < 0.001), where the annual refractive change gradually increased for children with mild myopia [−0.56 (−0.61, −0.50) D], moderate myopia [−0.65 (−0.71, −0.59) D], and high myopia [−0.73 (−0.81, −0.64) D] (F = 73.12, *P* < 0.001). There was no significant difference in the change of SE between children with hyperopia and children with mild myopia (F = 0.863, *P* = 0.422).

**Table 5:** Annual refractive change in children with different refractive statuses

	N (%)	Annual SE change in 2020	Annual SE change in 2021	t	*P*
All	12,766 (100%)	-0.68 (-0.69, -0.67)	-0.46 (-0.48, -0.43)	35.61	< 0.001
Hyperopia	4018 (31.47%)	-0.55 (-0.64, -0.50)	-0.53 (-0.63, -0.49)	20.94	< 0.001
Emmetropia	5674 (44.45%)	-0.49 (-0.52, -0.46)	-0.45 (-0.47, -0.41)	38.72	< 0.001
Mild myopia	1941 (15.20%)	-0.56 (-0.61, -0.50)	-0.55 (-0.58, -0.46)	24.56	< 0.001
Middle myopia	924 (7.24%)	-0.65 (-0.71, -0.59)	-0.62 (-0.66, -0.59)	25.36	< 0.001
High myopia	209 (1.64%)	-0.73 (-0.81, -0.64)	-0.78 (-0.82, -0.67)	17.73	< 0.001

### Factors affecting refractive error changes

Multiple regression analyses were performed with annual refractive change as the dependent variable and 15 risk factors of myopia progression as the independent variables (Table 6). The results showed that the younger the children (β=−0.045, *P* = 0.039), the female (β=−0.052, *P* = 0.018), the larger the BMI (β=−0.058, *P* = 0.016), the higher the baseline refraction (β=−0.053, *P* = 0.016), the lower the parental education level (β=−0.052, *P* = 0.031; β=−0.055, *P* = 0.021), and poorer parental intervention and eye care awareness (β=−0.036, *P* < 0.001) will show more myopic refractive change. In contrast, there was no significant correlation between birth gestational age, birth weight, parental myopia, frequency of eating sweets, time spent outdoors per day, time spent reading and writing homework per day, time spent using electronics per day, and type of electronics (all *P* > 0.05) and the amount of refractive error per year in children.

**Table 6:** Multivariate regression analysis of factors on refractive change of grade 1 to 3 students in Hubei Province

variable	Progression in 2020		Progression in 2021	
	β (95% CI)	*P*	β (95% CI)	*P*
Age	-0.045 (-0.053, -0.032)	0.039	-0.055 (-0.076, -0.041)	0.017
Sex	-0.052 (-0.057, -0.044)	0.018	-0.057 (-0.072, -0.039)	0.010
gestational age at birth	-0.018 (-0.025, -0.011)	0.440	-0.031 (-0.053, -0.026)	0.195
birth weight	-0.010 (-0.033, -0.005)	0.673	-0.034 (-0.053, -0.023)	0.120
body mass index (BMI)	-0.058 (0.049, 0.069)	0.016	-0.061 (0.048, 0.076)	0.006
Baseline refraction	-0.053 (-0.064, -0.037)	0.016	-0.106 (-0.133, -0.079)	< 0.001
Number of myopic parents	-0.047 (-0.058, -0.031)	0.348	-0.054 (-0.084, -0.033)	0.165
Father’s education level	-0.052 (-0.067, -0.035)	0.031	-0.076 (-0.091, -0.045)	< 0.001
Mather’s education level	-0.055 (-0.069, -0.033)	0.021	-0.113 (-0.153, -0.084)	< 0.001
Duration of outdoor activity each day	0.022 (0.013, 0.041)	0.330	0.074 (0.039, 0.092)	0.248
Duration of reading and writing each day	-0.030 (-0.043, -0.019)	0.656	-0.025 (-0.041, -0.018)	0.258
Duration of electronic devices each day	-0.031 (-0.043, -0.026)	0.160	-0.012 (-0.023, 0.024)	0.599
Type of electronics	-0.026 (-0.043, -0.019)	0.779	-0.029 (-0.031, -0.022)	0.195
Frequency of eating sweets	-0.089 (-0.097, -0.062)	0.094	-0.086 (-0.102, -0.057)	0.076
Parental intervention and eye-care awareness	-0.036 (0.013, 0.047)	< 0.001	-0.039 (0.023, 0.076)	< 0.001

### Predictive modeling of refractive error changes

**Fig. 1:**
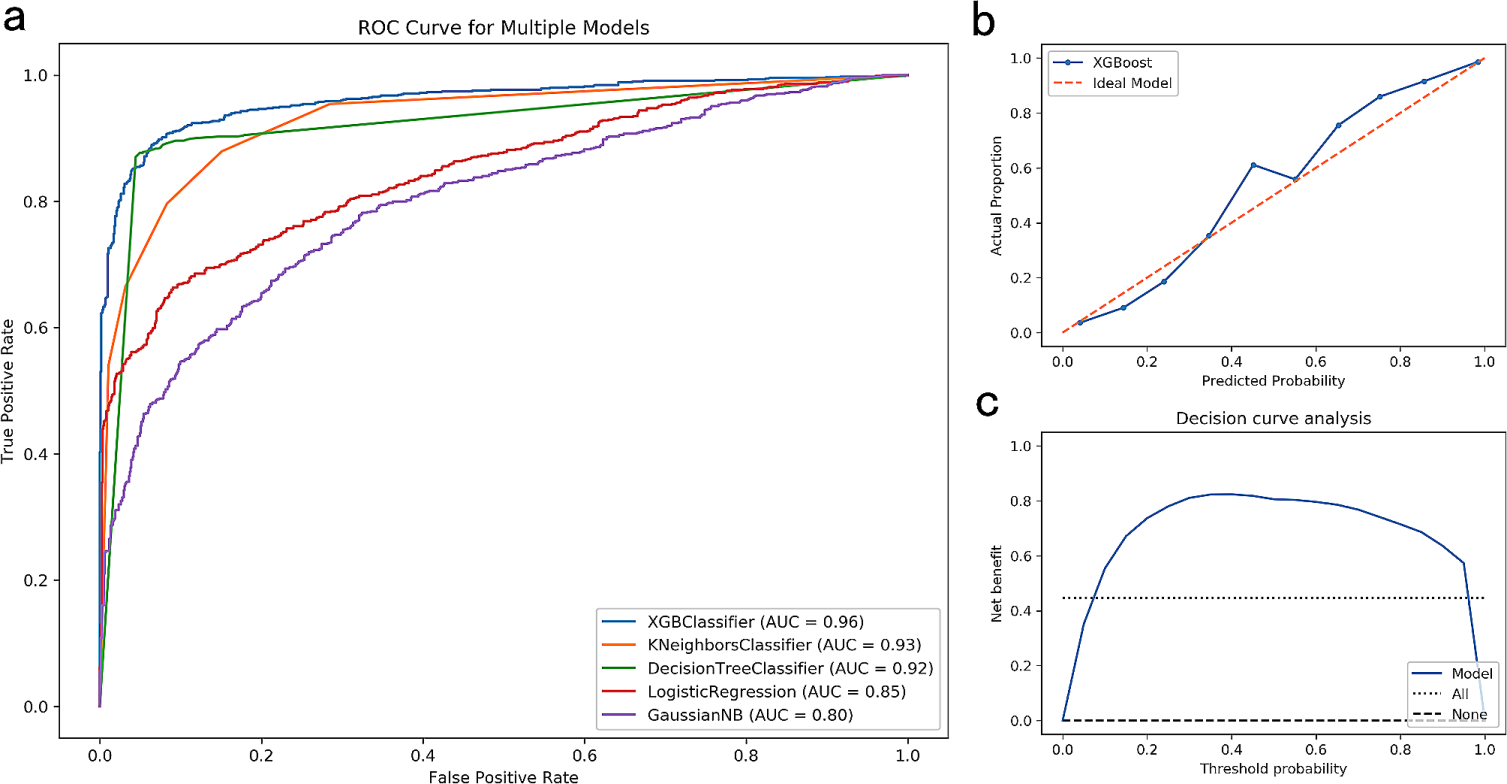
Prediction of the progression of myopia in children using the machine learning method. (a) ROC curves of refractive change predicted by 5 machine learning methods; (b) calibration curve of the XGBoost model; (c) decision curve of the XGBoost model

Five machine-learning methods were used to predict the change of SE every year for elementary school students in grades 1 to 3. In terms of predicting annual myopia progression, the area under curve (AUC)of the XGBoost model was 0.96 compared with the K Neighbors model (AUC = 0.93), Decision Tree model (AUC = 0.92), Logistic regression model (AUC = 0.85), and Gaussian NB model (AUC = 0.80), which showed that the XGBoost prediction model had good differentiation (Fig. 1. a). The DCA curve (Fig. 1. b) showed that the net benefit rates validated by the XGBoost model were high, and the CC curve (Fig. 1. c) verified that there was good agreement between the predicted value of the XGBoost model and the true value.

**Fig. 2:**
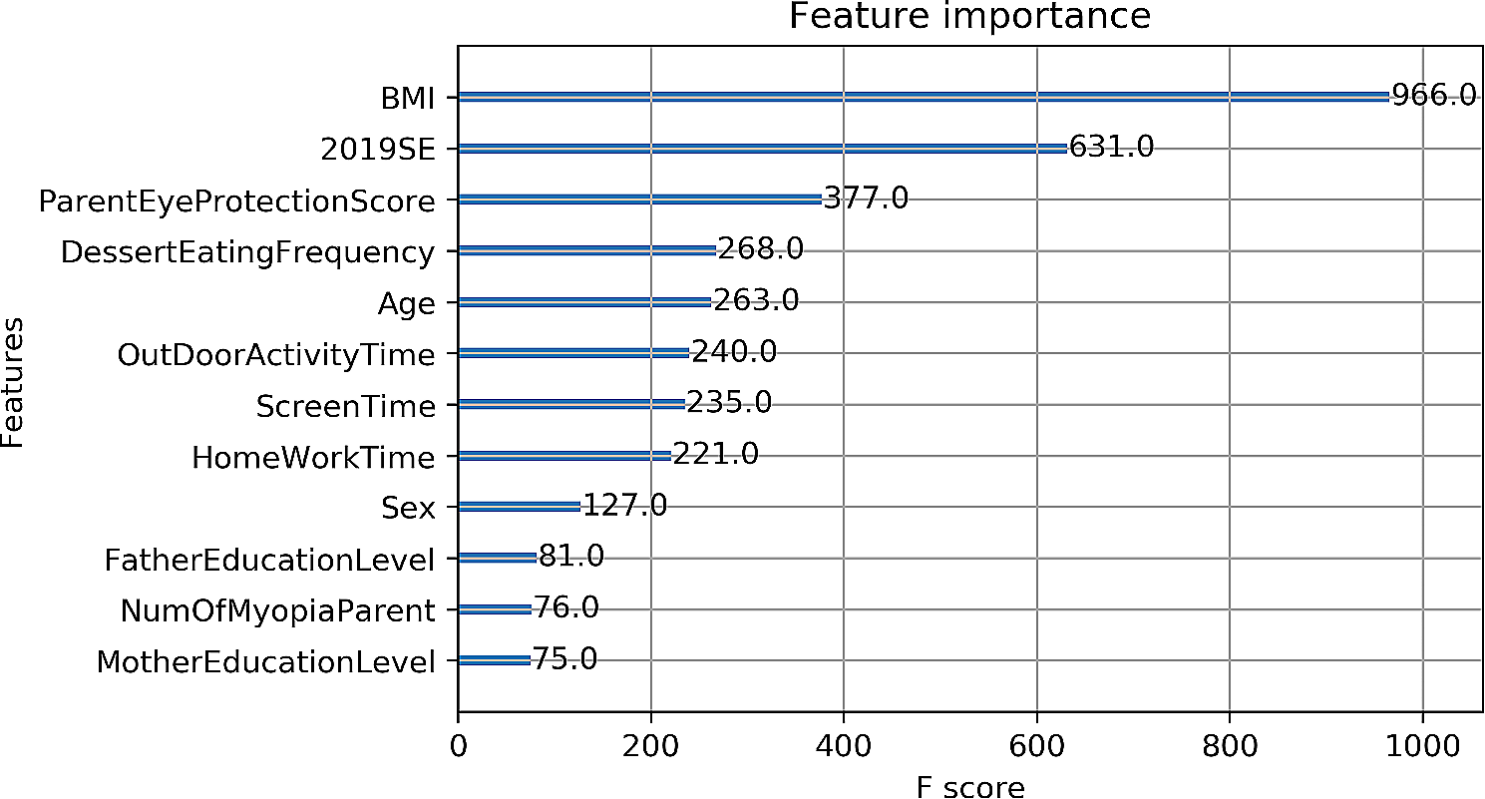
Analysis of the importance of each feature using the XGBoost model

The XGBoost model can demonstrate the degree of characteristic importance of each factor in the model prediction (Fig. 2). Children with a larger BMI also had a larger amount of refractive change per year thereafter, which was the most important influencing factor. Baseline refractive error was the second most important factor, with a larger baseline refractive error being associated with a faster refractive error change. This was followed by parental eye care awareness, frequency of eating sweets, age, time spent outdoors, daily hours of electronics, daily hours of reading and homework, gender, number of myopic parents, and parental education level.

**Fig. 3:**
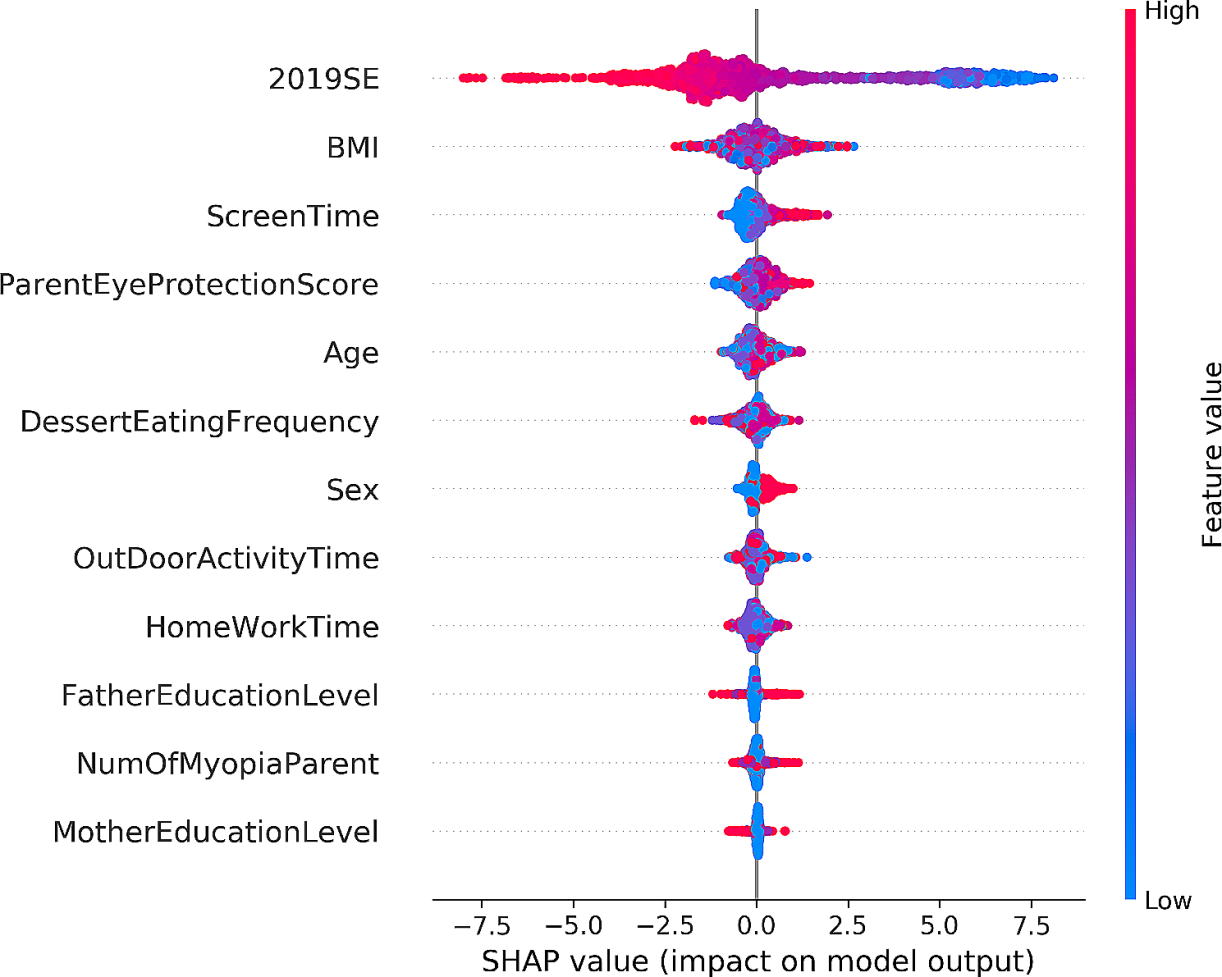
Beeswarm plots of the XGBoost prediction model of myopia progression

The SHAP method was further used to explain how the above influences affect changes in refraction. The SHAP values indicate the numerical value assigned to each feature in the model, with the absolute value reflecting the magnitude of the feature’s influence and the positive or negative reflecting its positive or negative effect. Figure 3 shows the risk contribution of the 10 features: higher baseline refraction (red) produces higher SHAP values, indicating that the more myopic the baseline refraction, the faster myopia progresses. Similarly, the risk of myopia progression increased for females, with longer electronic screen time and weaker parental eye care awareness. In contrast, the more time spent outdoors, the SHAP value increased as the value of this variable decreased (blue color), suggesting that outdoor activity is a protective factor in delaying myopia progression. The influence of other factors on myopia progression is unclear.

**Fig. 4:**
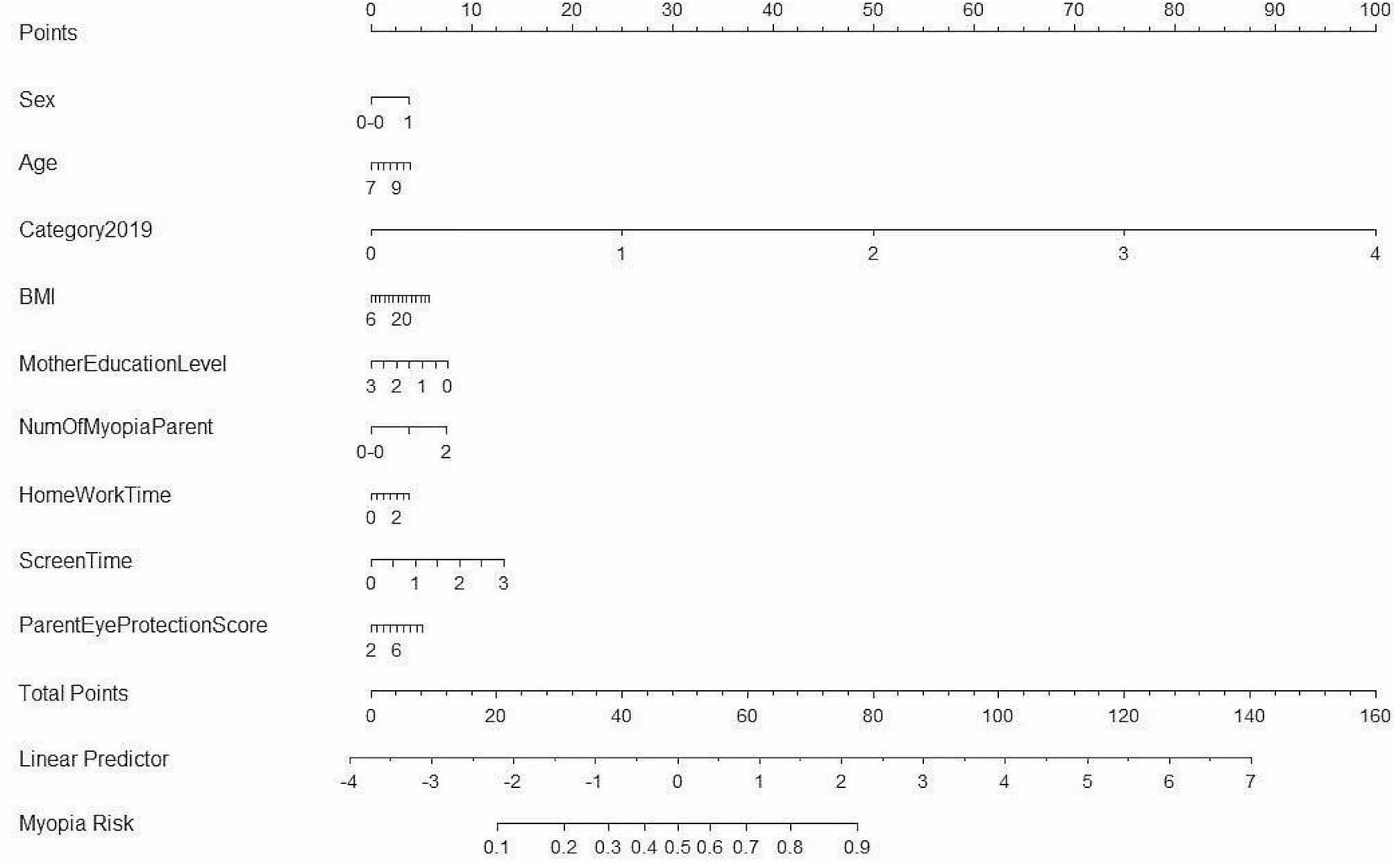
A nomogram to predict the probability of high myopia in grade 1 to 3 children in Hubei Province

The nomogram prediction model was based on the magnitude of the linear regression model correlation coefficients to develop a scoring scale that assigns a score to each of the various values of each influencing factor. A total score was produced based on each child’s baseline refractive status and myopia risk factor assignment, and the likelihood of outcome time occurrence for each patient was derived using the conversion function between the score and the probability of outcome occurrence. The axis structure and danger point indicated each variable’s effect and relevance on the expected result. Among the results of this study (Fig. 4), baseline refractive classification had the greatest predictive value, followed by electronic screen time, mother’s education level, number of myopic parents, BMI, and parental eye care awareness. By calculating the scores of each factor, the probability of developing high myopia in 7- to 10-year-old children in Hubei Province could be predicted. The total score of the model ranged from 0 to 160, corresponding to a risk rate of 0.1 to 0.9; the higher the total score, the higher the risk of developing high myopia.

## Discussion

Myopia progression is a process of an increase in spherical equivalent refraction. It is important to slow the progression of myopia in myopic children to avoid the development of high myopia and to minimize low vision and blindness due to complications of high myopia. As with the onset of myopia, myopia progression is a multifactorial process, with genetic and environmental factors playing independent roles and influencing each other. The amount of myopia progression varies by region, age, and sex. The mean age of the subjects in this study was 8.3 years, and the mean annual progression was − 0.68 D. It was similar to that in East Asian countries such as Hong Kong, China (−0.63 D) [15] and Singapore (−0.47 D) [16] and slightly higher than that in countries such as Australia (−0.31 D) [17] and the United Kingdom (−0.41 D) [18]. Cumulative progression at one, two, and three years of follow-up was higher in Asians than in Europeans − 0.31 D, −0.49 D, and − 0.58 D [19]. The slopes of the progression curves at different ages of onset are essentially parallel at each age, with faster progression at younger ages. Across the age range, the greatest annual change in refractive error was seen in children aged 6–10 years, and the smallest change was seen in adults aged 26–30 years. Children under the age of 15 exhibited much faster myopia advancement than those beyond the age of 15 [20]. If the age of onset of myopia is 7 years, the mean myopic progression in the following year is −0.58 D. For each additional year of age of onset, the annual progression of refractive error decreases by 0.07 D [21]. We found that the rate of progression of refraction was relatively faster in females than in males (*P* < 0.05). This may be due to the differences between male and female lifestyles [22], where females usually spend more time near work, such as reading and doing homework, which is considered a risk factor for myopia, and boys spend more time on outdoor activities, which is considered a protective factor against myopia. In addition, differences in sex hormone levels between males and females and the rate of structural changes in the body may also account for gender differences in refractive changes in SE [23].

We found a strong correlation between the rate of myopia progression and baseline refraction in 7- to 10-year-old students (*P* < 0.001). In the XGBoost prediction model, baseline refraction was the second most important influencing factor. The mean age of the study population in this paper was 8.30 ± 0.94 years, and myopia occurring at this age reflects more of a genetic susceptibility to myopia [24] and is relatively less influenced by environmental factors such as near work and outdoor activities. Lin et al. [25] found that the bigger the SE determined by baseline optometry, the more noticeable the change of refraction, and the myopia progression rate rose by 0.135D per year for every 1D rise in baseline SE. Hu et al. [26] found that preschool children with mild myopia at the initial visit exhibited a higher rate of myopia progression, which was the opposite of the pattern of change in school-age children, suggesting that factors associated with myopia progression in preschool children are different from those in school-age children. Verkicharla et al. [20] found that children with severe myopia (≤−9.00D) had myopic progression fastest, followed by high (<−6.00D to −9.00D), moderate, and low myopia. This might be because myopia evolves to the point where the biomechanical qualities of the scleral extracellular matrix are altered, increasing the likelihood of refractive error [27].

In this study, parental perception was investigated using a questionnaire, and the results showed that parental perception of myopia had a significant effect on the rate of myopia progression in their children (*P* < 0.05). In the XGBoost model, parental perception was the third factor after BMI and baseline refraction. Despite the current increasing prevalence of myopia in school-aged children, parents still have misconceptions about the dangers and treatment of myopia. For example, some parents refuse to fit their already myopic children with eyeglasses, believing that myopia grows faster with eyeglasses [28]. Choy et al. [29] surveyed 1,396 children and their parents in Hong Kong and found that only 23.6% of already myopic students’ parents were aware that their children had refractive errors, and only 19.8% wore glasses. Among the myopic children who did not wear glasses for correction, only 50% of the children had a log MAR VA < 0.2, and the number of children whose refractive errors were corrected by appropriate prescription lenses for the above children with a log MAR VA < 0.2 reached more than 85%. Beneficial parental vision interventions, such as limiting children’s prolonged near-work time (≥ 180 min/d) and prolonged use of electronic devices (≥ 60 min/d), can reduce the incidence of myopia in children, especially at elementary school [30]. Li et al. [31] conducted a two-year randomized clinical trial in Guangzhou, China, and found that weekly parental education about eye care through social media could significantly prevent and control the incidence of myopia. Health education through social media significantly prevented and controlled the onset of myopia in children while delaying the average annual progression of myopia by 0.25 D. In the long run, the beneficial vision intervention behaviors of parents influenced children’s behaviors and reduced the risk of children developing into high myopia, which is consistent with the results of the present study.

The progression of myopia is a multifactorial process. Traditional methods can analyze a limited number of influencing factors and do so with low precision. Machine learning uses algorithms to find associations between data, which can provide faster output and avoid confounding influences between factors. In recent years, machine learning techniques have demonstrated unique advantages in the assisted diagnosis of diabetic retinopathy [32], screening and diagnosis of glaucoma [33], and diagnostic grading and treatment plan selection for age-related macular degeneration [34]. Due to the stable development of various refractive elements in individuals at approximately 13 years of age, more accurate results can be obtained by applying their ocular parameters for prediction. In this study, we innovatively used various machine learning methods to construct a myopia progression prediction model and found that the most important influencing factors were BMI, baseline refractive error, parental eye care awareness, frequency of eating sweets, and age. Among them, large baseline refraction, large BMI, and high frequency of eating sweets were risk factors for myopia progression, while elder children and parental eye care awareness were protective factors for delaying myopia progression, effectively utilizing real-world data for the prevention and early diagnosis of high myopia. Higher BMI is significantly associated with high myopia but not with the prevalence of mild-to-moderate myopia in Korean children [35]. Similarly, an Australian twin study found that female myopes were heavier than those without myopia in weight, but there was no significant difference in BMI between the two [36]. Guan et al. [37] found that age, uncorrected distance visual acuity, and SE were predictors of the occurrence of high myopia in school-age children. Among them, age is an important factor because it implies higher cumulative educational pressure, which is consistent with the results of our study. Children with rapid initial refractive error progression may require closer monitoring and follow-up, as well as early clinical therapeutic intervention, as they are more likely to develop high myopia during growth and development. Therefore, our findings can help in risk stratification and guide clinical decision-making in the management of myopia prevention and control in children. For children at higher risk, more aggressive treatments, including a higher frequency of clinical follow-up, use of low-concentration atropine eye drops, or low-concentration atropine in combination with keratoplasty lenses, are needed to control the rate of myopia progression and to avoid progression to high myopia.

In conclusion, we found that the rate of myopia progression varied according to age, sex, and myopia severity among children in grades 1–3 in Hubei Province and that we need to focus on younger children, girls, and those with high myopia in myopia prevention and control. Machine learning methods can be used to build a prediction model for high myopia using real-world data. Large baseline refractive error, large BMI, and high frequency of eating sweets are risk factors for myopia progression, while high parental awareness of eye care is a protective factor to delay myopia progression, which needs to be confirmed by observation of a longer cohort study in the future to guide myopia prevention and control in clinical practice.

### Electronic supplementary material

Below is the link to the electronic supplementary material.

Supplementary Material 1

## Data Availability

The datasets generated and analyzed during the current study are not publicly available because of involving students’ privacy but are available from the corresponding author upon reasonable request.

## Abbreviations

VA: Visual acuity
SE: Spherical equivalent refraction
D: Diopters
BMI: Body mass index
CDC: Centers for Disease Control and Prevention
CI: Confidence interval
ROC: Receiver operating characteristic
CC: Calibration curve
DCA: Decision curve analysis
AUC: Area under the curve

## References

[1] Dolgin E (2015). The myopia boom. Nature.

[2] Morgan IG, French AN, Ashby RS, Guo X, Ding X, He M, Rose KA (2018). The epidemics of myopia: Aetiology and prevention. Prog Retin Eye Res.

[3] Dong L, Kang YK, Li Y, Wei WB, Jonas JB, PREVALENCE AND TIME TRENDS OF MYOPIA IN CHILDREN AND ADOLESCENTS IN CHINA (2020). A systemic review and Meta-analysis. Retina (Philadelphia Pa).

[4] Agarwal D, Saxena R, Gupta V, Mani K, Dhiman R, Bhardawaj A, Vashist P (2020). Prevalence of myopia in Indian school children: Meta-analysis of last four decades. PLoS ONE.

[5] Kobia-Acquah E, Flitcroft DI, Akowuah PK, Lingham G, Loughman J (2022). Regional variations and temporal trends of childhood myopia prevalence in Africa: a systematic review and meta-analysis. Ophthalmic Physiol Opt.

[6] Hagen LA, Gjelle JVB, Arnegard S, Pedersen HR, Gilson SJ, Baraas RC (2018). Prevalence and possible factors of myopia in Norwegian adolescents. Sci Rep.

[7] Schuster AK, Krause L, Kuchenbäcker C, Prütz F, Elflein HM, Pfeiffer N, Urschitz MS (2020). Prevalence and Time Trends in Myopia among children and adolescents. Deutsches Arzteblatt International.

[8] Harrington SC, Stack J, Saunders K, O’Dwyer V (2019). Refractive error and visual impairment in Ireland schoolchildren. Br J Ophthalmol.

[9] Rudnicka AR, Kapetanakis VV, Wathern AK, Logan NS, Gilmartin B, Whincup PH, Cook DG, Owen CG (2016). Global variations and time trends in the prevalence of childhood myopia, a systematic review and quantitative meta-analysis: implications for aetiology and early prevention. Br J Ophthalmol.

[10] Holden BA, Fricke TR, Wilson DA, Jong M, Naidoo KS, Sankaridurg P, Wong TY, Naduvilath TJ, Resnikoff S (2016). Global prevalence of myopia and high myopia and temporal trends from 2000 through 2050. Ophthalmology.

[11] Chen X, Ye G, Zhong Y, Jin L, Liang X, Zeng Y, Zheng Y, Lan M, Liu Y (2021). Prevalence, incidence, and risk factors for myopia among urban and rural children in southern China: protocol for a school-based cohort study. BMJ open.

[12] Morgan IG, Wu PC, Ostrin LA, Tideman JWL, Yam JC, Lan W, Baraas RC, He X, Sankaridurg P, Saw SM (2021). IMI Risk factors for myopia. Invest Ophthalmol Vis Sci.

[13] Cortina-Borja M, Smith AD, Combarros O, Lehmann DJ (2009). The synergy factor: a statistic to measure interactions in complex diseases. BMC Res Notes.

[14] Kuczmarski RJ, Ogden CL, Guo SS, Grummer-Strawn LM, Flegal KM, Mei Z, Wei R, Curtin LR, Roche AF, Johnson CL (2000). CDC Growth Charts for the United States: methods and development. Vital and Health Statistics Series 11 Data from the National Health Survey.

[15] Fan DS, Lam DS, Lam RF, Lau JT, Chong KS, Cheung EY, Lai RY, Chew SJ (2004). Prevalence, incidence, and progression of myopia of school children in Hong Kong. Invest Ophthalmol Vis Sci.

[16] Saw SM, Tong L, Chua WH, Chia KS, Koh D, Tan DT, Katz J (2005). Incidence and progression of myopia in Singaporean school children. Invest Ophthalmol Vis Sci.

[17] French AN, Morgan IG, Burlutsky G, Mitchell P, Rose KA (2013). Prevalence and 5- to 6-year incidence and progression of myopia and hyperopia in Australian schoolchildren. Ophthalmology.

[18] Wong K, Dahlmann-Noor A (2020). Myopia and its progression in children in London, UK: a retrospective evaluation. J Optometry.

[19] Donovan L, Sankaridurg P, Ho A, Naduvilath T, Smith EL 3rd, Holden BA. Myopia progression rates in urban children wearing single-vision spectacles. Optom Vis Sci. 2012;89(1):27–32. 10.1097/OPX.0b013e3182357f79 PMC3249020 21983120

[20] Verkicharla PK, Kammari P, Das AV (2020). Myopia progression varies with age and severity of myopia. PLoS ONE.

[21] Jones-Jordan LA, Sinnott LT, Chu RH, Cotter SA, Kleinstein RN, Manny RE, Mutti DO, Twelker JD, Zadnik K (2021). Myopia progression as a function of sex, Age, and ethnicity. Invest Ophthalmol Vis Sci.

[22] Grzybowski A, Kanclerz P, Tsubota K, Lanca C, Saw SM (2020). A review on the epidemiology of myopia in school children worldwide. BMC Ophthalmol.

[23] Chen ZT, Wang IJ, Liao YT, Shih YF, Lin LL (2011). Polymorphisms in steroidogenesis genes, sex steroid levels, and high myopia in the Taiwanese population. Mol Vis.

[24] Ip JM, Huynh SC, Robaei D, Rose KA, Morgan IG, Smith W, Kifley A, Mitchell P (2007). Ethnic differences in the impact of parental myopia: findings from a population-based study of 12-year-old Australian children. Invest Ophthalmol Vis Sci.

[25] Lin Z, Vasudevan B, Ciuffreda KJ, Zhou HJ, Mao GY, Wang NL, Liang YB (2017). The difference between cycloplegic and noncycloplegic autorefraction and its association with progression of refractive error in Beijing urban children. Ophthalmic Physiol Opt.

[26] Hu Y, Ding X, Long W, He M, Yang X (2019). Longitudinal changes in spherical equivalent refractive error among children with Preschool Myopia. Invest Ophthalmol Vis Sci.

[27] Boote C, Sigal IA, Grytz R, Hua Y, Nguyen TD, Girard MJA (2020). Scleral structure and biomechanics. Prog Retin Eye Res.

[28] Ang M, Flanagan JL, Wong CW, Müller A, Davis A, Keys D, Resnikoff S, Jong M, Wong TY, Sankaridurg P (2020). Review: myopia control strategies recommendations from the 2018 WHO/IAPB/BHVI meeting on myopia. Br J Ophthalmol.

[29] Choy BNK, You Q, Zhu MM, Lai JSM, Ng ALK, Wong IYH (2020). Prevalence and associations of myopia in Hong Kong primary school students. Jpn J Ophthalmol.

[30] Liu YL, Jhang JP, Hsiao CK, Tsai TH, Wang IJ (2022). Influence of parental behavior on myopigenic behaviors and risk of myopia: analysis of nationwide survey data in children aged 3 to 18 years. BMC Public Health.

[31] Li Q, Guo L, Zhang J, Zhao F, Hu Y, Guo Y, Du X, Zhang S, Yang X, Lu C (2021). Effect of School-Based Family Health Education via Social Media on Children’s myopia and parents’ awareness: a Randomized Clinical Trial. JAMA Ophthalmol.

[32] Ting DSW, Cheung CY, Lim G, Tan GSW, Quang ND, Gan A, Hamzah H, Garcia-Franco R, San Yeo IY, Lee SY (2017). Development and validation of a Deep Learning System for Diabetic Retinopathy and Related Eye diseases using retinal images from multiethnic populations with diabetes. JAMA.

[33] Asaoka R, Murata H, Hirasawa K, Fujino Y, Matsuura M, Miki A, Kanamoto T, Ikeda Y, Mori K, Iwase A (2019). Using deep learning and transfer learning to accurately diagnose early-onset Glaucoma from Macular Optical Coherence Tomography images. Am J Ophthalmol.

[34] Peng Y, Dharssi S, Chen Q, Keenan TD, Agrón E, Wong WT, Chew EY, Lu Z (2019). DeepSeeNet: a Deep Learning Model for Automated classification of patient-based Age-related Macular Degeneration Severity from Color Fundus photographs. Ophthalmology.

[35] Lee S, Lee HJ, Lee KG, Kim J (2022). Obesity and high myopia in children and adolescents: Korea National Health and Nutrition Examination Survey. PLoS ONE.

[36] Dirani M, Islam A, Baird PN (2008). Body stature and myopia-the genes in myopia (GEM) twin study. Ophthalmic Epidemiol.

[37] Guan J, Zhu Y, Hu Q, Ma S, Mu J, Li Z, Fang D, Zhuo X, Guan H, Sun Q (2023). Prevalence patterns and onset prediction of high myopia for children and adolescents in Southern China via Real-World Screening Data: Retrospective School-based study. J Med Internet Res.

