# Supplementary material for: Study of myopia progression and risk factors in Hubei children aged 7–10 years using machine learning: a longitudinal cohort
Source: BMC Ophthalmol. 2024 Mar 1;24:93. doi: 10.1186/s12886-024-03331-x (PMC10905806; doi:10.1186/s12886-024-03331-x)
Supplement: Supplementary file 1 — Supplementary Material 1 [file 12886_2024_3331_MOESM1_ESM.docx]

**Appendix 1**

**Questionnaire on students' eye habits and myopia-influencing factors**

Dear parents,

We’d like to invite you to complete the following questionnaire that aims to investigate students' eye use behavior and myopia-influencing factors. The personal information involved in the questionnaire will be kept strictly confidential, please feel free to fill it in.

Thank you very much for your support and cooperation!

Myopia Prevention and Control Center for Children and Adolescents in Hubei

Department of Ophthalmology, Renmin Hospital of Wuhan University

**School______________ Class__________ Name_________ Gender_________ Age________**

**Date of completion ______Y______M______D**

**Part 1 Basic information**

|  | **Birth History** | | |
| --- | --- | --- | --- |
| **A1** | Was the child born | 1. Full term (≥37 weeks) 2. Pre-term (<37 weeks) | |
| **A2** | Birth weight | _________ kg | |
| **A3** | Mode of delivery | 1. spontaneous delivery 2. cesarean delivery | |
|  | **Information regarding the parents** | | |
|  | | **Father** | **Mother** |
| **B1** | Age | ______Years old | ______Years old |
| **B2** | Educational level | 1. Junior middle school equivalent or less  2. Senior middle school equivalent (Technical school)  3. Undergraduate degree (Junior college/college)  4. Postgraduate degree (Masters, PhD) | 1. Junior middle school equivalent or less  2. Senior middle school equivalent (Technical school)  3. Undergraduate degree (Junior college/college)  4. Postgraduate degree (Masters, PhD) |
| **B3** | Has myopia？ | 1. Yes **(If Yes, please answer question B4)** 2. No 3. Don’t know | 1. Yes **(If Yes, please answer question B4)**  2. No  3. Don’t know |
| **B4** | Degree of myopia | 1. Mild myopia (＜3.00D)  2. Middle myopia (3.00～6.00D)  3. High myopia (＞6.00D)  4. Don’t know | 1. Mild myopia (＜3.00D)  2. Middle myopia (3.00～6.00D)  3. High myopia (＞6.00D)  4. Don’t know |

**Part 2 Eating and sleeping habits**

Please recall your child’s eating and sleeping habits listed below in **the last 7 days**, and choose according to the actual situation of the child or ask the child's true experience

|  | **Questions** | **The general situation in the last 7 days** |
| --- | --- | --- |
| **C1** | Your child is picky about food? | 1. No 2. Yes |
| **C2** | How many times did your child drink carbonated beverages in the last 7 days? | 1. Never 2. Once or twice a week  3. 3~5 times a week 4. Every day |
| **C3** | How many times did your child eat sweets (such as chocolate, milk tea, ice cream, etc.) in the last 7 days? | 1. Never 2. Once or twice a week  3. 3~5 times a week 4. Every day |
| **C4** | Time your child goes to bed each night | 1. Earlier than 20:30 2. 20:30~20:59  3. 21:00~21:29 4. 21:30~21:59  5. Later than 22:00 |
| **C5** | Time your child wakes up each day | 1. Earlier than 6:30 2. 6:30~6:59  3. 7:00~7:29 4. 7:30~7:59  5. Later than 8:00 |
| **C6** | Does your child sleep with the light on? | 1. Never 2. Sometimes  3. Often 4. Always |
| **C7** | How long does your child usually take a nap? | 1. Never 2. Less than 0.5 h  3. 0.5~1 h 4. More than 1 h |

**Part 3 Eye-using habits**

Please recall your child’s eye-using behavior listed below in **the last 7 days**, and choose according to the actual situation of the child or ask the child's true experience

|  | **Questions** | **The general situation in the last 7 days** |
| --- | --- | --- |
| **D1** | How long does the child spend outdoors during the day？ | 1. Less than 1 h 2. 1~2 h  3. 2~3 h 4. More than 3 h |
| **D2** | How long does the child spend reading books and doing homework every day? | 1. Less than 1 h 2. 1~2 h  3. 2~3 h 4. More than 3 h |
| **D3** | Does the child rest his / her eyes after a period of continuous reading? | 1. Yes 2. No |
| **D4** | How long does the child spend using electronic devices (tablets, phones, computers, etc.) every day? | 1. Less than 1 h 2. 1~2 h  3. 2~3 h 4. More than 3 h |
| **D5** | What types of electronic devices do the children use the most every day? | 1. Television 2. Computer  3. Phone 4. Tablet |
| **D6** | Adequate light when reading and writing | 1. Never 2. Sometimes  3. Often 4. Always |

**Part 4 Myopia prevention and treatment:** please tick accordingly.

|  | **Questions** | **Choices** |
| --- | --- | --- |
| **E1** | Does your child have myopia? | 1. Yes 2. No 3. Don’t know |
| **E2** | Any other eye conditions (such as cataracts, premature retinopathy, etc)? | 1. No 2. Yes, _________ |
| **E3** | Parents' knowledge of vision care | 1. Exactly not 2. A little  3. Some 4. Very well |
| **E4** | Whether to take the child to the hospital every six months to check eyesight. | 1. Yes 2. No |
| **E5** | Do parents urge children to protect their eyes? | 1. Never 2. Sometimes  3. Often 4. Always |
| **E6** | Whether to wear glasses for children after myopia | 1. Yes 2. No, reason: _________ |
| **E7** | What do you think of the harm degree of myopia to the eyes? | 1. Light 2. Moderate 3. Heavy 4. severe |
